# A Proposal of a Combined Convergence Regulatory Strategy Applied to Post-approval Changes by Latin American Countries, Reducing Workload and Allowing Continuous Improvement to Guarantee the Quality, Safety, and Efficacy of Medicines

**DOI:** 10.3389/fmed.2021.768376

**Published:** 2021-11-23

**Authors:** Heraclio Rodriguez, Maria Lucia De Lucia

**Affiliations:** ^1^Head of Global Regulatory Science and Policy for Latin America, Sanofi, Panama; ^2^Lead of Global Regulatory Science and Policy for Latin America, Sanofi, Panama

**Keywords:** post-approval changes, reliance, recognition, national regulatory agencies, quality risk management

## Abstract

In recent years, post-approval changes (PACs) for medicinal products have increased faster than the national regulatory agencies can attend to without causing any negative impact. This study presents a proposal for regulatory management based on our analysis of the data available from the national regulatory agencies of Latin America on the total post-approval changes evaluated, and the time spent in the process. A retrospective search on the official websites of competent national regulatory authorities (NRAs) of 14 Latin American countries (México, Guatemala, Nicaragua, Honduras, El Salvador, Panamá, Costa Rica, Venezuela, Colombia, Ecuador, Peru, Argentina, Chile and Brazil) was conducted to collect data on post-approval changes in the last 4–6 years, up to January 2021. The NRAs considered were Brazil, México, Colombia, and Costa Rica. Our analysis was focused on the post-approval changes that required approval before implementation, those that were submitted, and those that were submitted and approved for small molecules, biologics, and biotechnological products. The results indicated differences in the regulatory processes and procedures applied by the different agencies. We also found that the implementation of the PACs was directly impacted by limited resources, which puts the medication supply for chronic treatments at risk resulting in serious consequences for patients. For local decision-making, Latin American NRAs should implement regulatory pathways already made by regulatory agencies included in the World Health Organization Listed Authorities on PAC approval to optimize their resources and to ensure the continuity of medicine supply for their patients.

## Introduction

Chemistry, Manufacturing, and Controls (CMC) changes in medicinal products are inevitable regardless of their type, category, or characteristics. They can be observed in the form of a technological transfer to the final manufacturer of the products during the developmental phase, changing technical needs due to new findings during the product lifecycle, and continuous improvement in the manufacturing processes and product characteristics (1).

Thus, regulations demand a careful evaluation of all changes and proper follow up in the context of regulatory pathways, regardless of whether it is a drug under investigation or a commercial product. To guarantee the quality, safety, and efficacy of the product, leveraging both product and process knowledge as well as the use of a risk-based approach should allow sponsors to achieve the best path for post-approval change (PAC) implementation and regulators to optimize resources through accelerated regulatory pathways (2).

In a recently published report by the Pan American Health Organization (PAHO) (3), it was declared that the marketing authorization in the Latin American national regulatory authorities (NRAs) of regional reference is a complex area, which poses a number of challenges for regulators at present and will continue to do so in the future. The NRAs tend to devote a significant share of staff resources to marketing authorization. However, growing markets will generate more associated lifecycle demands (4). As a result, the number of PACs submitted has piled up through the years, creating a large backlog that can take a significant amount of time to be cleared by even the largest and most well-funded authorities (3). This challenge demands more resources from all NRAs (who must be using their limited resources efficiently), considering that CMC processes are crucial in guaranteeing the optimal quality, safety, and efficacy of the medicines distributed in their countries. One pathway that should be covered in order to achieve optimization would be through the regulatory reliance on the assessment and approvals performed and granted by the Stringent Regulatory Authorities of the product's manufacturing countries [described in the World Health Organization (WHO) List of Stringent Regulatory Authorities].

The WHO, which finds and fosters the best capabilities of NRAs to promote the standardization concept and its principles around the world, has raised the need for reviewing the classification level of regulatory agencies. For this reason, it issued a robust and unique version of the “WHO Global Benchmarking Tool” for the evaluation of the national regulatory systems of medical products (5). It also evaluates and publicly designates regulatory authorities as WHO Listed Authorities (WLA) after going through a more demanding process (6). WHO developed these guidelines in response to the barriers and gaps that impact the regulatory systems, cause inefficiency, and limit access to safe, effective, and quality medical products.

The designation of a regulatory authority as a WLA is ultimately meant to promote access to the supply of safe, effective, and quality medical products by facilitating reliance on the work and decisions of trusted agencies in the regulatory decision-making process to reduce the extra work and wastage of limited financial resources.

In this context, an NRA receives the classification Level 4 (this is the NRA with regulatory systems operating at an advanced level of performance and continuous improvement) (6), if its regulatory processes, evaluations, and decision-making fall within Good Regulatory Practices (7) based on the nine principles: legality, consistency, independence, impartiality, proportionality, flexibility, clarity, efficiency, and transparency. The NRA should also have a robust and well-functioning quality management system (QMS). This system includes the application of quality risk management (QRM) principles to support regulatory authorities in achieving greater credibility for their decisions. QMS contributes to systematic planning, control, and improved quality in all processes throughout all the regulatory functions and ensures a comprehensive approach for all the processes involved (7).

For its part, PAHO (8) had previously recognized eight national regulatory authorities of regional reference (NRAr) based on its own tool. In 2019 it recognized the National Administration of Drugs, Foods and Medical Devices (ANMAT) of Argentina; the National Health Surveillance Agency (ANVISA) of Brazil; the Center for State Control of Drugs, Equipment, and Medical Devices of Cuba; the Federal Commission for Protection against Health Risks (COFEPRIS) of México; Health Canada; the Public Health Institute (ISP) of Chile; the National Food and Drug Surveillance Institute (INVIMA) of Colombia; and the United States Food and Drug Administration (US FDA) in this context. Some of them have started exchanging information related to good manufacturing practices through a virtual platform known as the “Regulatory Exchange Platform–Secure.”

By considering risk quality management an essential part of good manufacturing practice along with other related guidelines, this proposal aims to consider the possible mechanisms that can be implemented among Latin American countries. Our primary purpose is to provide recommendations for the more efficient management of the PACs and ensure the planned supply flow of pharmaceutical (small molecules), biological, and biotechnological products in the NRAs of less mature countries. Specifically, for this proposal, statistical data related to PACs and posted on the NRAs websites of four Latin American countries (Brazil, México, Colombia, and Costa Rica), were extracted and analyzed. One of them is an active member of the International Council for Standardization of Technical Requirements for Pharmaceuticals for Human Use (ICH) Management Committee (Brazil), and two others are ICH Observers (México and Colombia). This analysis was supported by the regulatory framework and the criteria for classification of PACs, in the countries of the Stringent Regulatory Agencies, based on risk quality management.

For several years, some of Latin America's NRAs have maintained dynamic control and improved their processes, performing routine data collection and publishing reports related to the volume of submissions and ongoing internal processes approved, delivered, or rejected, which involves new registrations and the PACs.

However, not all countries in the region have available data on their official websites. There were discrepancies found among health agencies in how the data are registered as well as in their reporting periods because some authorities report for a period of 3–4 years, while others report the data every year; this poses a challenge that should be overcome when conducting an objective analysis.

Under this scenario, 4 (ANVISA-Brazil, COFEPRIS-México, INVIMA-Colombia and Ministry of Health-Costa Rica) out of 14 agencies were selected, with the available data related to PACs.

To encourage Latin American NRAs to optimize their resources and ensure the continuity of medicine supply for their patients, we also aimed to implement regulatory pathways such as the recognition of local decision-making by the regulatory agencies already included in the WLA concerning the issue of PAC approval. Our approach is based on QRM applied by the manufacturer, confirmed during the good manufacturing practices (GMP) inspection under strict compliance with the guidelines of the WHO and the ICH, which guarantees efficacy, safety, and supply of medicine.

## Materials and Methods

To perform this analysis, the official websites of competent NRAs from 14 Latin American countries (México, Guatemala, Nicaragua, Honduras, El Salvador, Panamá, Costa Rica, Venezuela, Colombia, Ecuador, Peru, Argentina, Chile, Brazil) were systematically reviewed, and a retrospective search of available data related to PACs evaluated or submitted during the last 4–6 years until January 2021 was done. After reviewing and validating the data with a Sanofi Regulatory team from each country and experts searching for information on the websites, the agencies to be included in this analysis were, specifically, ANVISA-Brazil, COFEPRIS-México, INVIMA, and Ministry of Health-Costa Rica.

It is important to mention that the Caribbean Islands were not included in this search of data reported.

Since common criteria in the available data collected from these regulatory agencies was not found (neither in the definition of the type of product nor in PAC classification), the analysis focused on:

PACs that require approval before implementation

PACs submitted

PACs submitted and approved

Synthetic, biologic, and biotechnological products

In this sense, the detailed information used for the analysis is as follows:

### ANVISA-Brazil

Classification of PAC: Minor and Major

Type of products (ANVISA definition): Synthetic, Generics, Technology-Biologics, Biologics

Data available: (9) Total PACs evaluated, and Time (days) reported from 2016 to 2021^*^.

Period: 2016-Jan 2021

^*^This period is the last period updated available in the ANVISA website up until January 2021.

### COFEPRIS-México

The data available was collected only from 2011 until 2016 since there was no data reported after 2016 (10). That is why we only included in this analysis data collected during 2014, 2015 and 2016.

Type of product (COFEPRIS definition): IV (sale under medical prescription), VI (sale Over the Counter).

Data available: PACs approved are reported annually.

Target Time for evaluation: 45 days (11).

### INVIMA-Colombia: (12)

There was no data on PACs submitted for public use as evaluated and approved in Colombia.

Thus, our analysis was based on the Ministry of Health and Social Protection report involving PACs evaluated by INVIMA from 2000 until 2020.

Data used: PAC submission data extracted from the Ministry of Health and Social Protection report (12).

Type of product: Not defined

Period: 2000–2020

### Costa Rica-Ministry of Health: (13)

Type of products: Small molecules and biologics

Data available: PACs submitted per year

Period: 2017–2020

Target Time for evaluation: (13) Small molecules-73 days; biological products-62 days

For each country involved, the data analyzed was the following:

Total number of PACs processed or submitted during the study period, according to the data collected by each regulatory agency.

Average time of assessment after submission annually. We considered a scenario of uniform distribution of the PACs between the years of data collection (considering months consisting of 20 working days) to calculate the number of months required by the regulatory authority to complete the assessment before implementation of the PAC.

The focus of this analysis was the calculation of the total number of PACs evaluated per year and the time (in days and months) spent by each NRA to process the PACs. Our analysis also evaluated how the current PAC process impacted the implementation of continuous improvement required to guarantee the quality, safety, and efficacy of pharmaceutical products and their supply. With the exception of ANVISA, all countries included in this analysis did not differentiate between PACs Ia/Ib or lower and PACs type II or higher (according to EMA and FDA classification). There was also a delay in implementing PACs that only require notification in the country of manufacture until emission and reception of approval from the regulatory authorities of the Latin American country where it is commercialized.

Our analysis is complemented by the assessment of publicly available regulations and guidelines regarding the requirements to guarantee the products' quality, efficacy, and safety as a fundamental support for regulatory decision-making.

## Results

### ANVISA-Brazil

Period of Analysis: January 2016–January 2021 (9)

ANVISA started data collection in 2016 by classifying PACs as minor and major for synthetic (small drugs), generic, and biological products, similarly to the EMA and FDA classifications. ANVISA has registered a particular classification specifically for biological products, named “technologic biologic products” (Biotechnology) which has been quantified as a major PAC category for this analysis.

**Figure 1:**
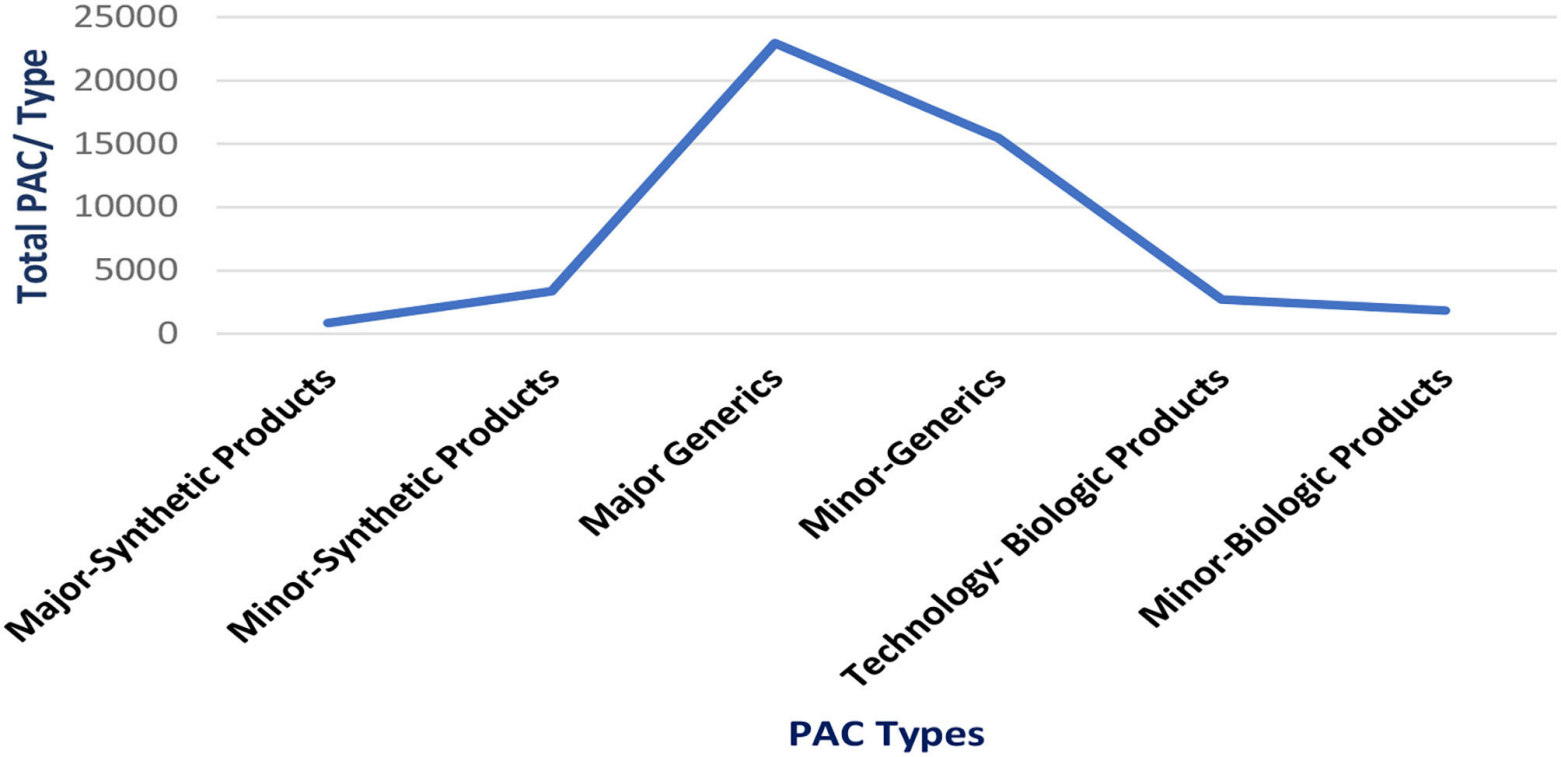
Post-approval changes processed in Brazil (2016−2020).

Until January 2021, a total of 47.182 PACs was evaluated (most of them generic products), with a time of 2,953 days invested by the regulatory agency team. Please see Figure 1.

**Figure 2:**
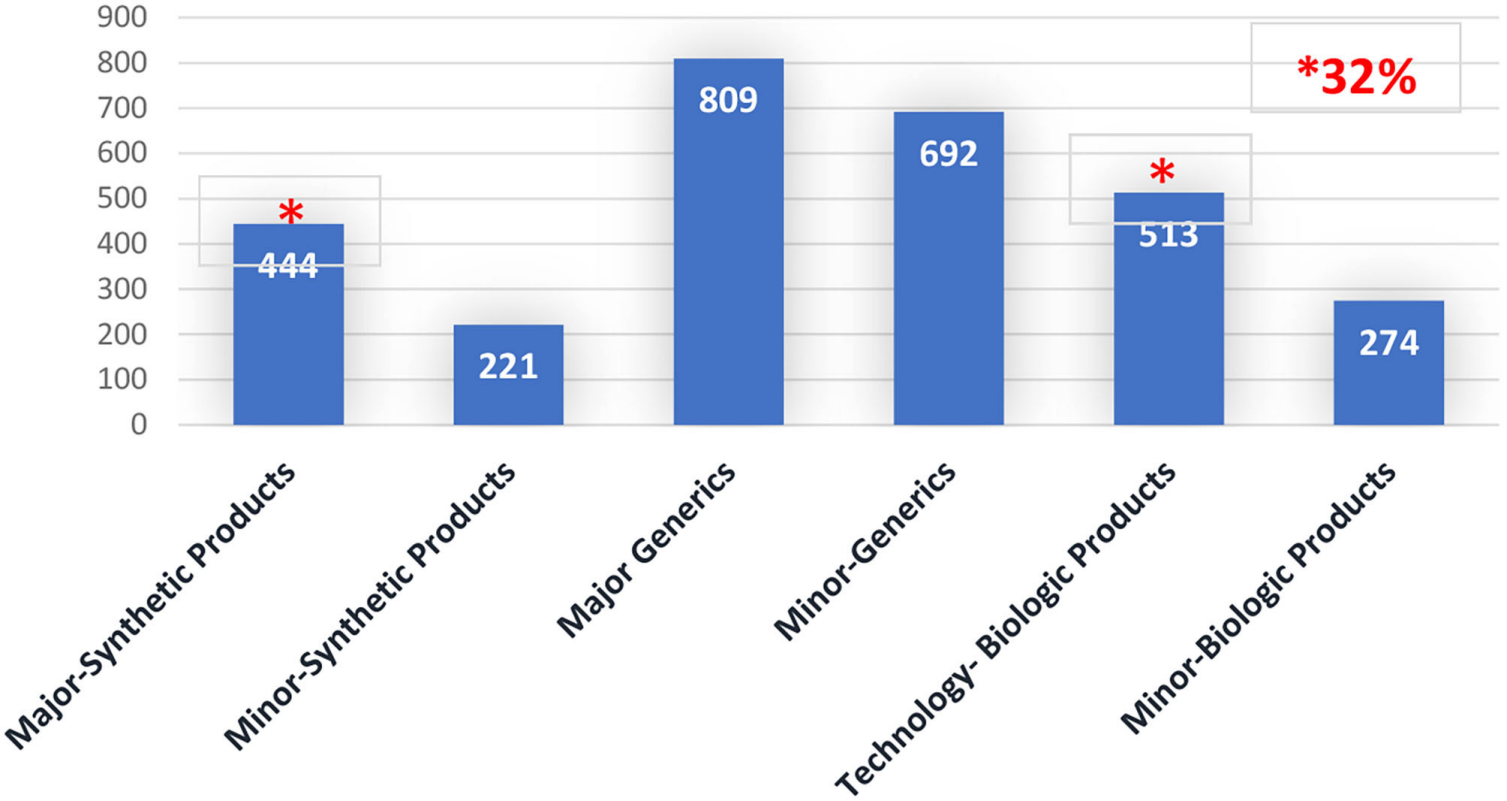
Post-approval changes timeline (Days), per PAC type by product's category. Brazil 2016–2020.

When the time spent assessing major PACs per category of the product is analyzed, 15% was spent on major synthetic products (small drugs) (total 444 days) while 17% was spent on technologic biologic products (Biotechnology) (total 513 days). Please see Figure 2.

Considering that a month has 20 workdays, approximately 47.85 months is necessary for assessing major PACs, distributed across 22.2 months for major PACs of synthetic products and 25.65 months for technologic biologic products.

### COFEPRIS-México

Period of Analysis: 2014–2016 (10)

Data collected was related to products classified in categories IV and VI, with Category IV products approved to be sold only with a medical prescription (Rx) and Category VI products approved to be sold Over the Counter.

A total of 1272 PACs was approved during these years (~424 per year), with a significant percentage of PACs being approved related to Category IV products used to treat several types of chronic diseases. Even though there is little information related to the time spent for approval or on the kind of products included in group IV, neither data was available. Concerning the submissions processed after 2016, we can infer that the number of PACs submitted has increased over the years considering the new marketing authorization issued by COFEPRIS as well as the continuous improvement of processes during the lifecycle of sold products.

**Figure 3:**
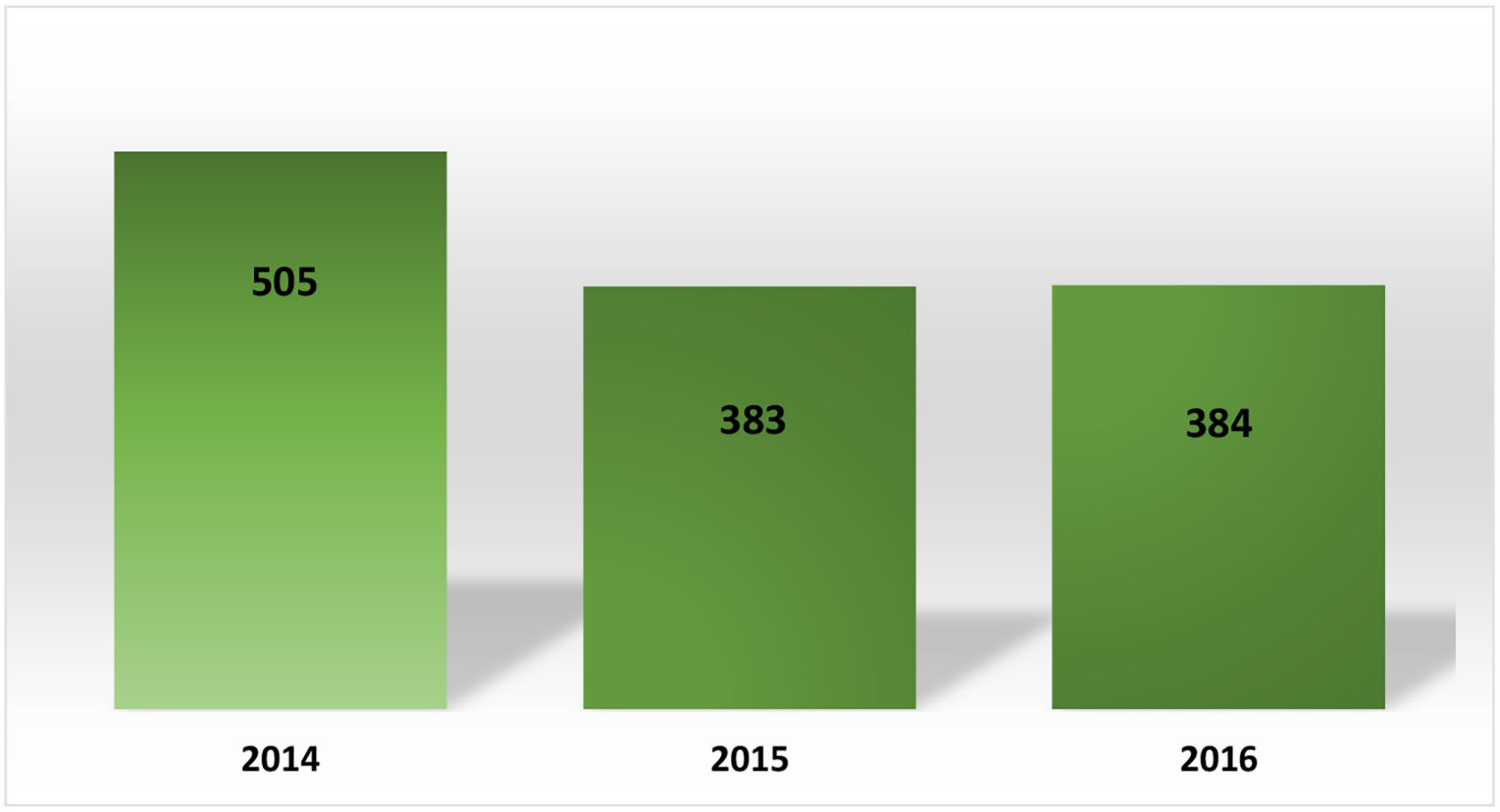
Post-approval changes processed in Mexico (2014–2016).

**Figure 4:**
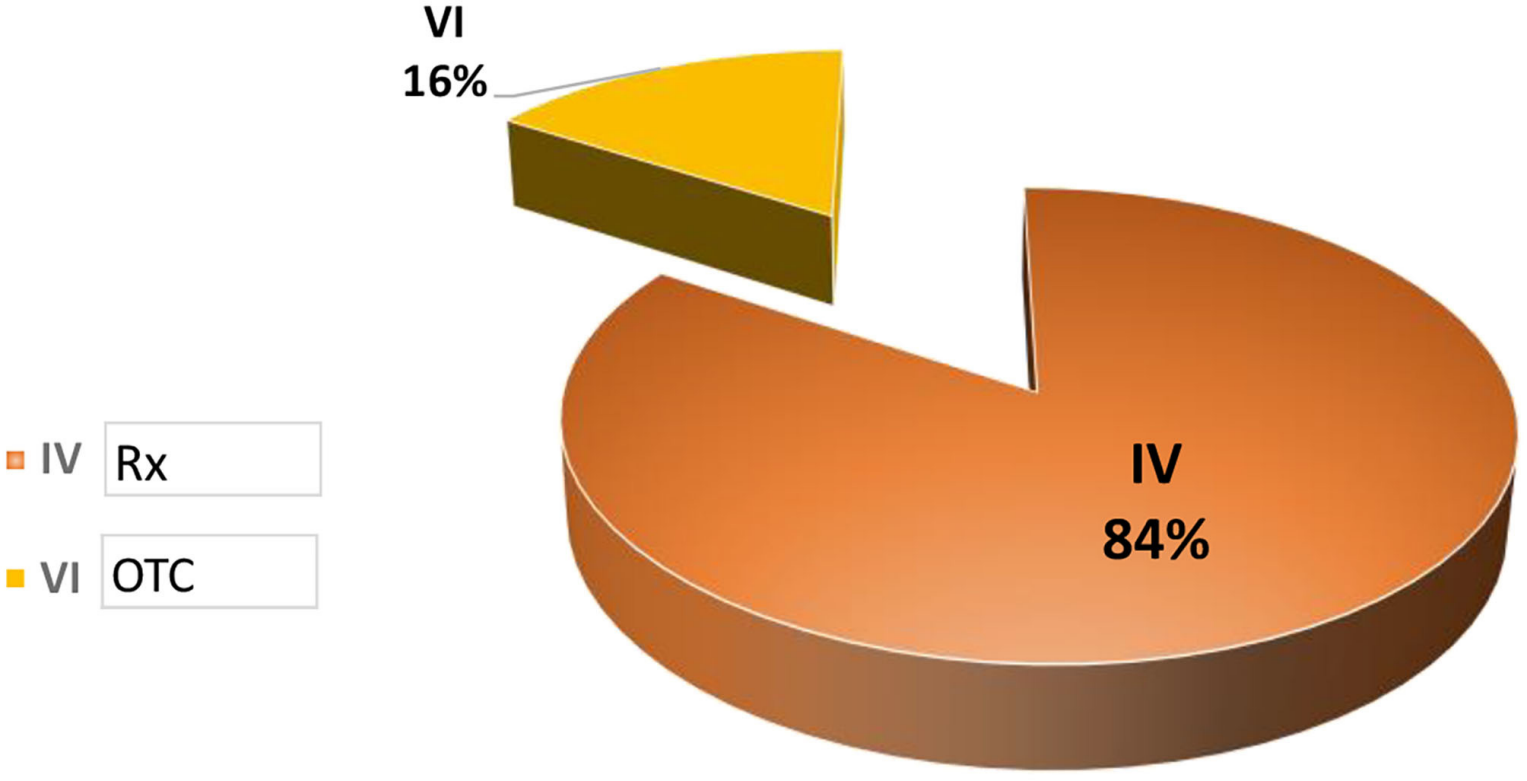
Post-approval changes processed in México.

It is important to highlight that AMIIF (a Mexican trade association) (11) reported on its website that there were 441 PACs waiting for approval during the time that the data was analyzed. Based on the average per year calculated with data collected by COFEPRIS during 2014–2016 (~424 per year), we can consider that the COFEPRIS spent ~1 year of work on PAC matters, with a possible negative impact on the implementation of these PACs and the supply of these therapies. Please see Figures 3, 4.

### INVIMA-Colombia

Period of Analysis: 2000–2020

The Ministry of Health and Social Protection (along with INVIMA) published a final report on the evaluation of the regulation reviewed, with regards to Decree 677 published in 1995 (12).

**Figure 5:**
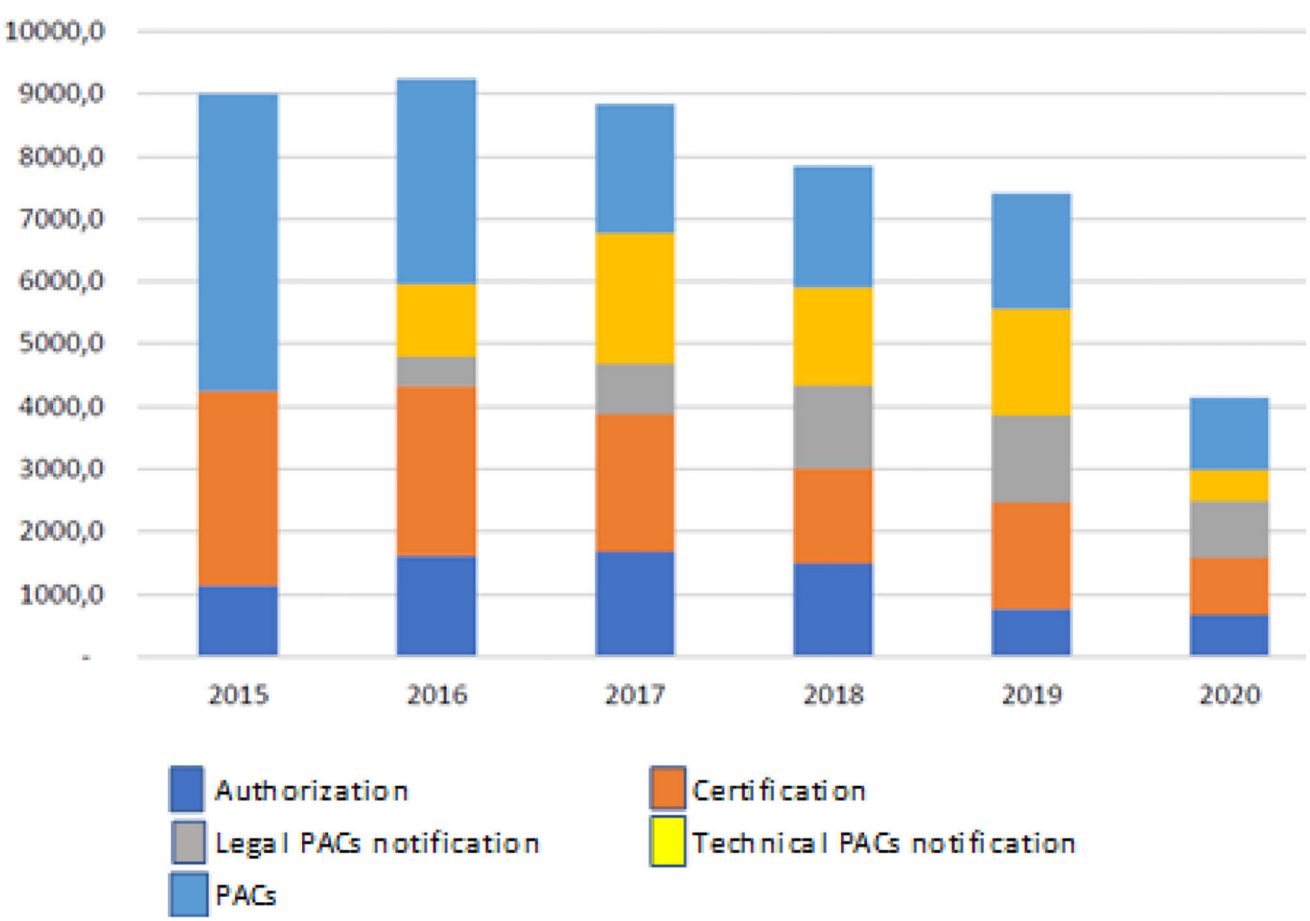
Modification, authorization, and certification of requests for small molecules in Colombia 2015–2020. Source: Invima 2020.

This report documents the results obtained after assessment by the Ministry of Health, INVIMA, and the World Bank on the current regulations. The results indicate that a total of 36,319 submissions (comprising new products and renewals) were processed during the analyzed period, with new products comprising 62.7% (22,780 submissions) of the total. This report also highlights an increase in PAC's submission throughout the years, which overloaded the INVIMA and impacted the availability of products, as we can see in Figure 5, taken from the report (12).

### Costa Rica-Ministry of Health

Period of Analysis: 2017–2020 (13)

**Figure 6:**
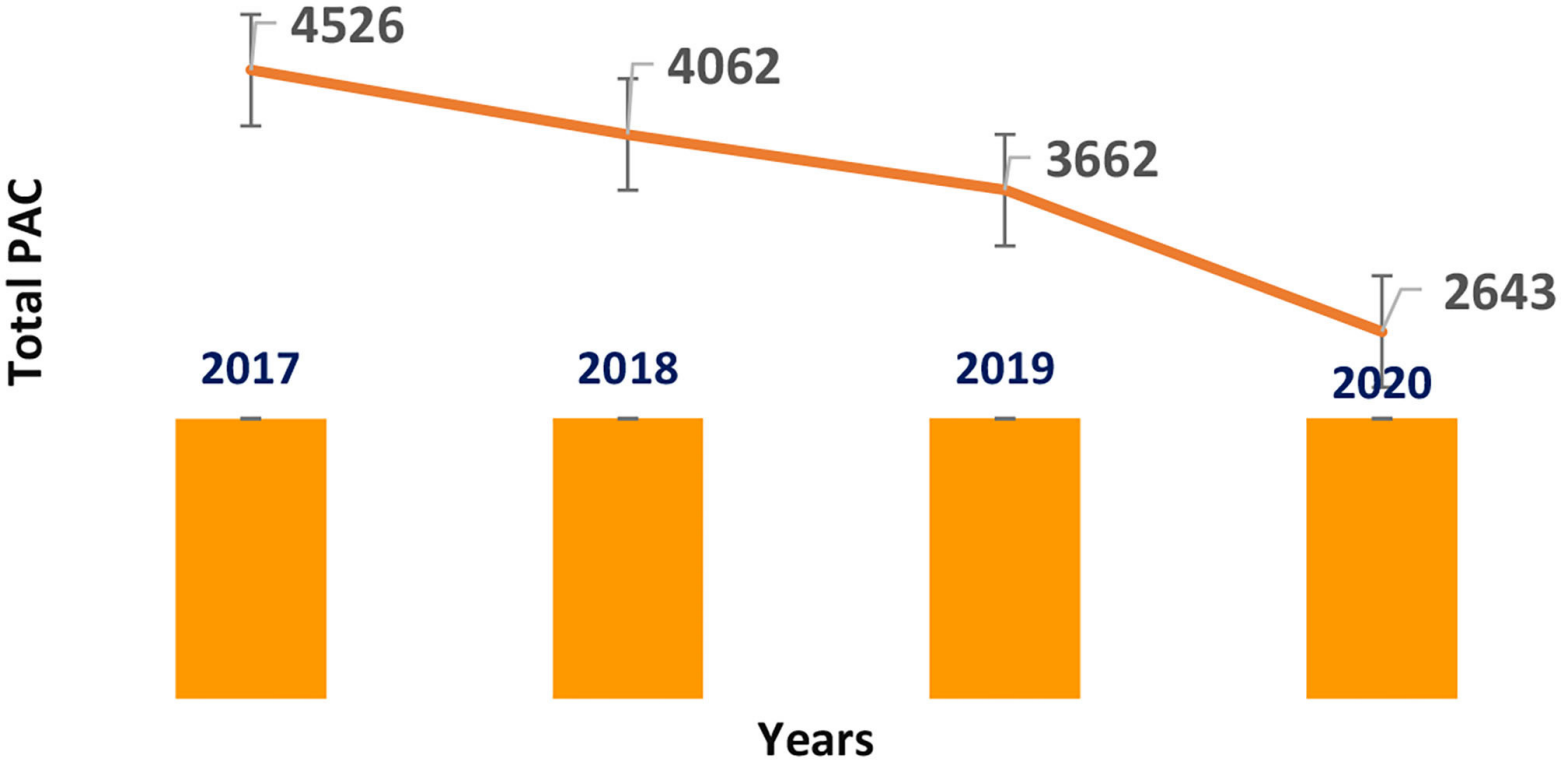
Post-approval changes submitted in Costa Rica (2017–2020) small molecules.

Costa Rica is a member of the Regulation Technical Committee of Central America (*Reglamento Técnico Centro América*). According to the RTCA 11.03.64:11, PACs are classified into two types: one is related to the changes that require approval from the NRA before implementation (it involves a majority of PACs related to quality, efficacy and safety), while the other is related to changes that only require the NRA to be notified concerning their implementation, which comprises primarily minor issues like the change of material/dimensions of the secondary packaging, change of the primary and secondary package label design, discontinuations of registered presentations, changes in the product safety information, and the change or broadening of the distributors. Please see Figure 6.

After reviewing the last 4 years, a total of 16,269 PACs on small molecules and Biologics products were evaluated.

**Figure 7:**
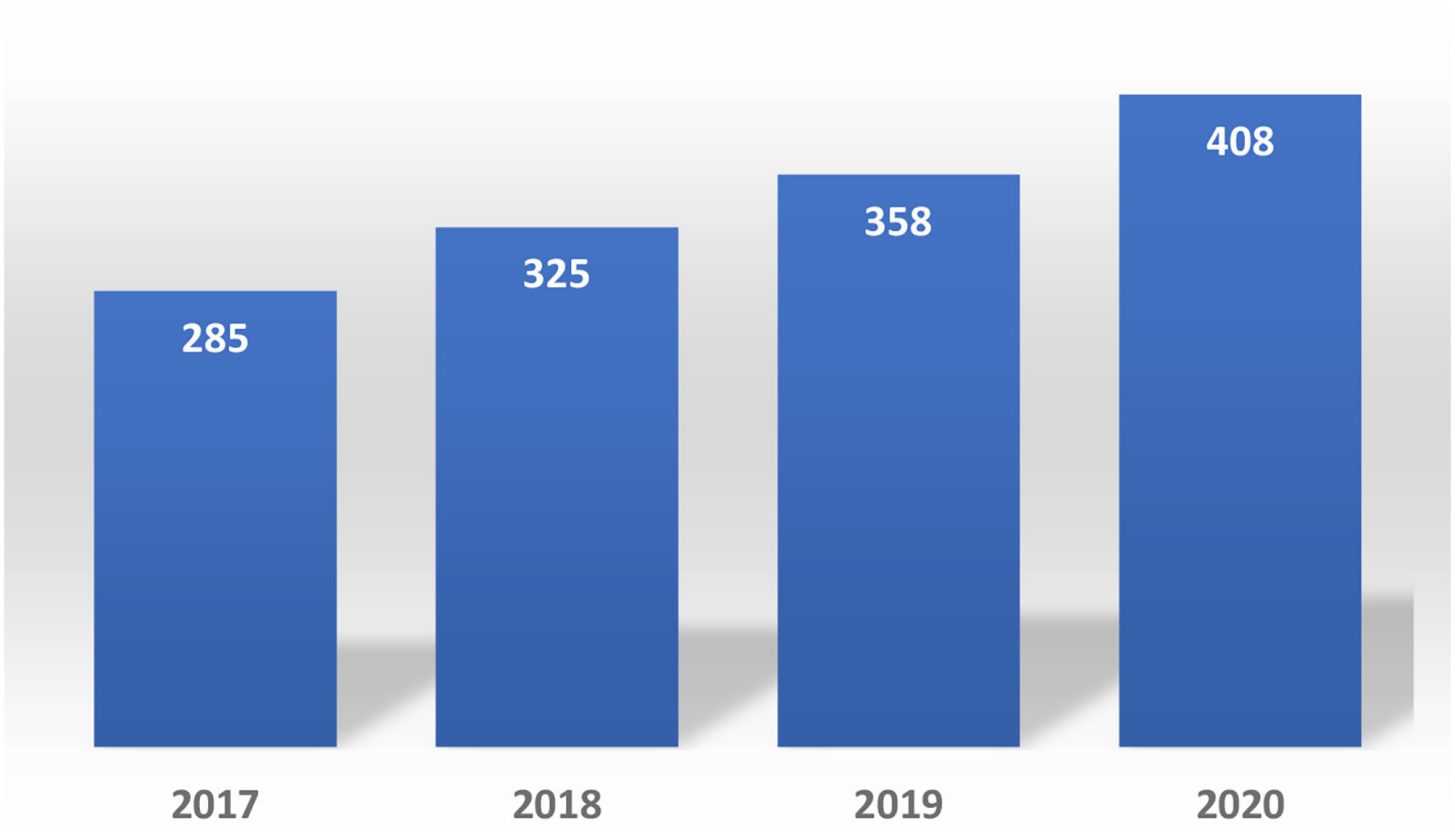
Post-approval changes submitted in Costa Rica (2017–2020) biologics.

In 2017, the NRA of this country processed more than 4,500 PACs of small drugs, while they processed more than 3,600 PACs in 2019. The average time spent assessing PACs related to technical information in this group of products was 73 days per submission (14), 13 days more than the timeline required by the regulations. Please see Figure 7.

With regards to the biologic products, the number of PACs submitted in 2020 was 43.15% higher compared to 2018 and 13.96% higher compared to 2019. Each PAC required an average of 62 days to be completely assessed.

## Discussion

The results obtained clearly show a need to optimize regulatory mechanisms and procedures applied in Latin America by NRAs regarding the management of PACs. They should consider the QRM system implemented by the Pharmaceutical Industry and the Stringent Regulatory Authorities, as an essential factor in maintaining good quality, safety, and efficacy on the basis of the regulatory framework described as follows.

## Basic Principles to Guarantee the Quality, Efficacy, and Safety of Pharmaceutical Products included in the Regulatory Framework Supporting The Issuance Of Good Manufacturing Practice Certificates And The Criteria To Classify And Evaluate PACs For The Regulatory Agencies Included In The WLA

Good Manufacturing Practices are based on the QMS and QRM according to regulations established by WHO and ICH. They help ensure the main of quality, safety, and efficacy parameters that all medicinal products should meet. We will review some documents on the subject as issued by them.

### World Health Organization (WHO)

The 54th meeting of the WHO Expert Committee on Specifications for Pharmaceutical Preparations (ECSPP) was held in Geneva, Switzerland, from 14 to 18 October 2019. Annex 5 on QMS requirements for national inspectorates is defined as follows: (15).

#### Quality Management System

An appropriate infrastructure encompassing the organizational structure, procedures, processes, resources, and systematic actions necessaries to show robust evidence documented to ensure confidence with regards to a product or service to satisfy requirements for quality.

A documented change management system should be established to ensure that changes requests are assessed, approved, or rejected; that appropriate resources are allocated; and roles and responsibilities defined. Any change should be documented, communicated to the personnel, and evaluated after implementation, to ensure objectives are met. The change management system should ensure that continuous improvement is undertaken in a timely and effective manner.

It is also important to highlight that appropriate quality indicators and methods should be established to monitor and periodically evaluate the inspectorate processes and level of improvement and service (including contracted-out services) to demonstrate that they were performed as planned and they have met the parameters predefined as the Fifty-fourth Report Quality Objectives in WHO Technical Report Series No. 1025 by the Expert Committee on Specifications for Pharmaceutical Preparations (15). These quality indicators, methods, analyses, and results should be documented. The results of the analyses should be used to evaluate the performance and effectiveness of the QMS, the adequacy of actions taken to address risks, and the need for further improvement.

#### Annex 2: WHO Good Manufacturing Practices for Pharmaceutical Products: Main Principles: This Document Mentions the Following

1. Quality management is a wide-ranging concept covering all matters that individually or collectively influence the quality of a product. It is the totality of the arrangements made with the object of ensuring that pharmaceutical products are of the quality required for their intended use. Quality management, therefore, incorporates GMP and other factors, including those outside the scope of this guide, such as product design and development (16).
2. GMP Is Aimed Primarily at Managing and Minimizing the Risks Inherent in Pharmaceutical Manufacturing to Ensure the Quality, Safety, and Efficacy of Products.

#### Annex 3: WHO Good Manufacturing Practices for Biological Products This Document Highlights the Following

1. The concepts of QA, GMP, QC, and QRM (17) are interrelated aspects of quality management and should be the responsibility of all personnel.
2. The system of QA appropriate to the manufacture of pharmaceutical products should ensure that there is a system for approving changes that may impact product quality. Regular evaluations of the quality of pharmaceutical products should also be conducted to verify the consistency of the process and ensure its continuous improvement; there is also a system for QRM.

Changes are an essential part of the lifecycle of the products in constant improvement. That is why the WHO considers the need for manufacturing sites to have an appropriate QMS to ensure the QRM receives the GMP Certificate.

### International Council for Standardization of Technical Requirements on Pharmaceuticals for Human Use (ICH)

#### Guidelines ICH Q9 and ICH Q10

According to the ICH Q9 (18), QRM can be applied to different aspects of pharmaceutical quality. These aspects include not only development, manufacturing, and distribution, but also the inspection and submission/review processes throughout the lifecycle of drug substances, drug (medicinal) products, biological, and biotechnological products on the use of raw materials, solvents, excipients, packaging and labeling materials.

ICH Q10 (19) mentions that the use of “QRM” can improve the decision-making processes from development, technical transfer, and manufacturing to PACs, and throughout the entire product life cycle. The QRM is strongly linked to the concept of Knowledge Management, where the Quality Target Product Profile (QTPP) is defined, including Critical Quality Attributes during the design and Critical Process Parameters (CPP) in the manufacturing process design, to identify and predict all possible variations occurring during and after the escalation to commercial batches.

#### Guideline ICH Q12

This guideline demonstrates how an increase in production and process knowledge can contribute to a more precise and accurate understanding of which PACs require regulatory submission and emphasizes the importance of an effective pharmaceutical quality system in the management of changes during the product lifecycle. Such management will eventually reduce unnecessary expenses and time burdens on the industry and regulators. In the meantime, reliable access to high quality medicinal products for patients is assured while continuous improvement is supported. This may result in decreased variability of products and in increased manufacturing efficiency. Implementation of this guideline can also mitigate drug shortages related to manufacturing and quality issues and facilitate the introduction of innovations in manufacturing.

ICH has been ensuring access to therapies while guaranteeing, through the guidelines issued, the quality, safety, and efficacy of drugs. They also minimize the risks in each step involved from the development through the clinical investigation, from manufacture until the final product use.

Applying ICH Q9, Q10, and Q12 (20) principles, as proposed by the One-Voice-of-Quality Group (the Chief Quality Officers of 25 multi-national pharmaceutical companies) (2) should enable pharmaceutical companies to report to regulators only the PACs which really need to be assessed. If manufacturers can demonstrate that they have an effective QMS for managing PACs (as defined by PIC/s), many PACs can be managed internally without waiting for prior approval from regulators.

Standardizing the classification of reporting categories by creating a “notification” category where it does not already exist is also important. This move would enable regulators to be informed of minor or moderate PACs, as defined under ICH Q12, and avoid delaying the implementation of such PACs by ensuring they are not mistakenly classified as major PACs. It would also ensure appropriate consistency with the way PACs are managed by the Stringent Regulatory Authorities.

In this sense, a common understanding and application of QRM principles could facilitate mutual confidence and promote more consistent decisions among regulators, based on the same information. This collaboration could be important in developing policies and guidelines integrating and supporting QRM practices.

It is important to mention that participation in international standardization and convergence initiatives can help to strengthen regulatory systems (3).

A good example of this would be the EMA and FDA because both NRAs have based their regulations on the QRM and adopting PAC classification according to the risk level for health and the impact on the quality, safety, and efficacy of the medicinal products, as well as implementing an annual system of notification regarding modifications classified as minor importance. This process can be implemented without previous approval of what has happened during this period (particularly by the EMA) based on the mutual recognition principle, which establishes that the evaluation of a variation that requires approval from some countries members must be done by one of the NRA involved and that the assessment and decision be adopted by the other regulatory authorities to reduce work duplication (21, 22).

Another important forum is the Pharmaceutical Inspection Co-operation Scheme (PIC/S), which supports regulatory inspections by developing common standards in the field of GMP and ensuring that those standards are consistently implemented across their jurisdictions (3).

### Proposals

The use of reliance (23) or recognition in the assessment and decision-making on the PACs by the Stringent regulatory agency in the country of origin can be a good practice of convergent strategies recommended to NRAs of Latin American countries to improve the efficiency of the regulatory processes implemented during the lifecycle of the pharmaceutical products. This would guarantee the quality, safety and efficacy of therapies, minimizing the shortage of medications, and ensuring the required access for all patients.

**Figure 8:**
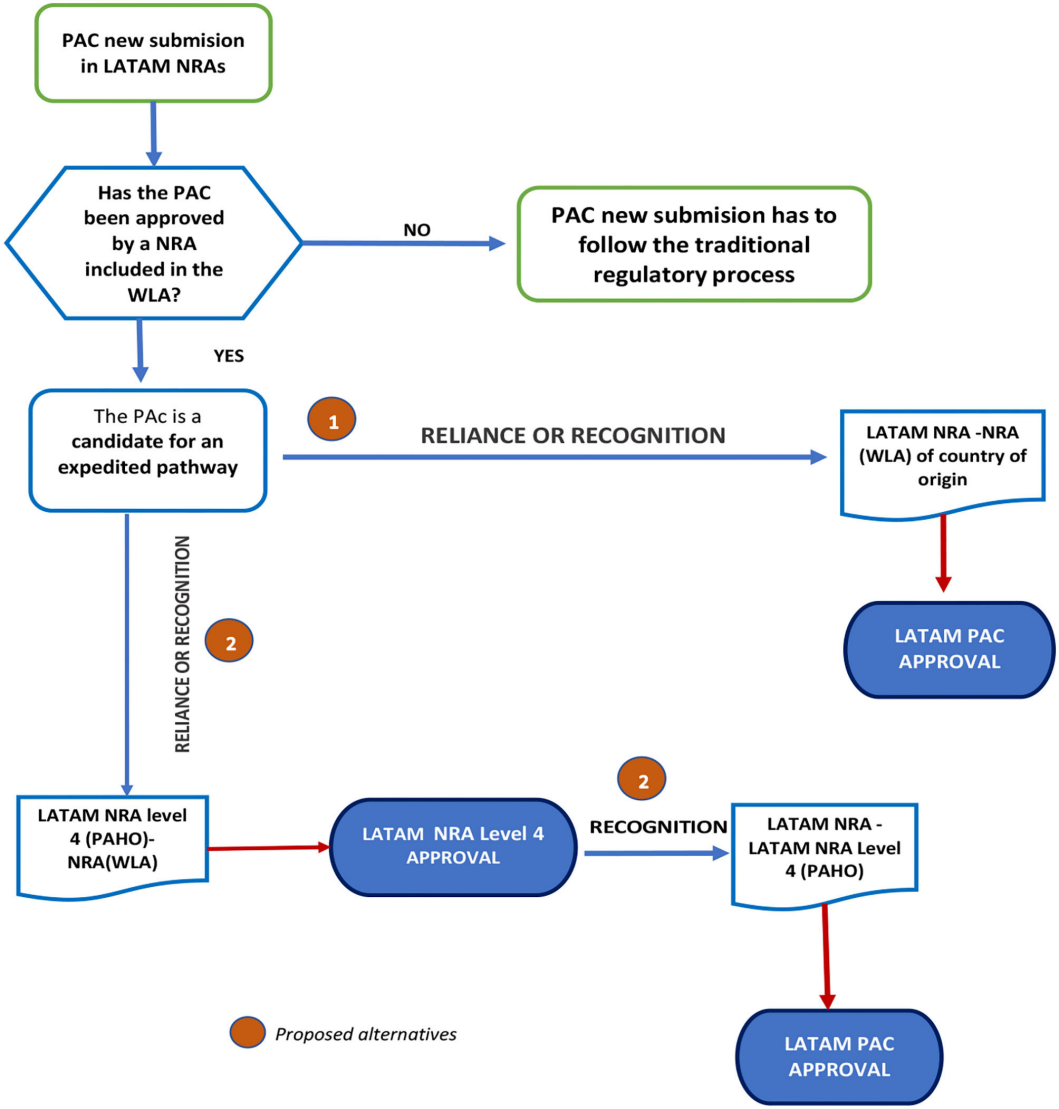
Proposed flowchart for applying reliance or recognition for PACs submitted for prior approval.

Considering the mechanisms or agreements established for local regulations, the proposal consists of the following steps:

1. The LATAM NRAs adopt reliance or recognition from the Stringent Regulatory Agencies (SRA) in the respective country of origins.
2. Regional Strategy: once the PAC is approved following the reliance or recognition process by a Level 4 LATAM NRA according to PAHO classifications, the other NRAs in the region could follow suit. Please see Figure 8.

The basis of this suggestion is the capacity of the regulatory authority in the country of origin to validate and certify implementation and proper functioning of the effective pharmaceutical quality system (involving the QRM) according to recommendations established in the ICH and WHO Guidelines. This suggestion also considers that COFEPRIS-MÉXICO, ANVISA-Brazil, INVIMA-Colombia, ISPE-Chile, and ANMAT-Argentina are regulatory agencies recognized as NRAr by PAHO in Latin America.

The principle of recognition mentioned is supported and described in the REGLAMENT (EC) No 1234/2008 OF THE COMMISSION OF NOV 24th, 2008: Commission REGLAMENT (EC) No 1234/2008 concerns the examination of variations to the terms of marketing authorization for medicinal products for human and veterinary use (21), to reduce work duplication in case the PAC requires approval from more than one country member.

Reliance promotes a more efficient approach to regulation, thereby improving access to quality-assured, effective, and safe medical products. It can take many forms and can be applied to varying degrees while recognizing or considering assessments, decisions, or authorized information from other authorities and institutions (23).

This proposal can only be implemented if the product approved and sold in each Latin American country has the same formula, manufacturing process, specifications, and analytical method certified by the person responsible for the product's manufacture.

By adapting these elements into the Latin America NRA regulations, resources and energy can be focused on the most important PACs while PACs with minor to moderate potential impact on the product quality, safety and efficacy can be processed much quicker.

We confirmed the differences in the regulatory processes and procedures among the NRAs through the data collected from the NRAs in Brazil, Colombia, México, and Costa Rica. We also looked at how limited resources directly impact the implementation of the PACs, which puts the medication supply for chronic treatments at risk and subsequently results in serious consequences for patients.

Benefits obtained through the implementation of these proposals:

- *Patient Benefits:*

° Availability of therapies, and a lower risk of back orders which ensures a continuous supply of crucial medicines.
° Facilitation of access to innovative therapies.
° Treatments with compliant quality, safety, and efficacy within established parameters.

- *LATAM Reference regulatory agency Benefits*:

° Process optimization.
° Improvement of timeline approval and implementation times.
° Updated real-time dossiers aligned with the manufacturing country.
° Increased of technical and scientific capabilities
° Standardization of criteria related to PACs with Stringent Regulatory Agency.

- *LATAM NRAs Agency Benefits:*

° Optimization of resources used in other areas of interest such as anti-counterfeiting and pharmacovigilance.
° Updated real-time dossier aligned with the manufacturer and the NRA used as reference.
° Quality dossier standardization among the countries.
° Standardization of criteria related to PACs around the region.

- *Health System Benefits:*

° Reduction in shortage of critical therapies (oncology, antibiotics, chronic therapies).
° Timely implementation of improved therapies with high quality, efficacy, and safety.

- *Pharmaceutical Industry benefits*.

° Manufacture unification by product.
° Reduction of time needed to implement changes required to improve the quality, safety, and efficacy of the product.
° Ensure timely supply of the product for each country and reduces shortages and discontinuation of treatment for patients.

## Conclusion

In this analysis, we systematically reviewed all available NRA official websites of LATAM countries. A potential limitation of this analysis is the availability of PAC data from all NRA websites. At the same time, some discrepancies among countries were found in the data collected, which requires more attention from NRAs in the region.

We observed clear differences in the regulatory frameworks of different NRAs in Latin American countries vs. the agencies included in the WLA (like the EMA and the FDA) with regards to PAC classification. PACs classified as a minor variation or Type Ia and Ib (by the FDA and EMA) can be implemented immediately or after 30 days, respectively, by the manufacturer. However, the situation is different in Latin America because once PACs are approved by stringent agencies, some NRAs must wait for approval or authorization which impacts the manufacturers, and distribution centers, as well as causing other supply issues.

Therefore, globally standardized and consistent regulatory approaches to PACs as proposed in the WHO's guidance on variations (24), along with clear and consistent timelines for assessment and approval of these PACs should lead to improved predictability to manage them. There should also be an improvement in resource-saving, a decrease of complexity in managing global supply chains, a reduction of the risk for drug shortages, and encouragement for companies to adopt innovative technology to supply drugs manufactured with the highest quality standards (25, 26).

Despite discrepancies in the data available, the analysis shows that the time spent by NRAs per year to evaluate PACs should be considered a key performance indicator to evaluate NRAs efficiency in terms of resource management. They can define a target timeline for approval (not exceeding 6 months) without a negative impact on the improvements required to guarantee the quality, safety, and efficacy of pharmaceutical products and their supply in the region.

Some recommendations proposed by PAHO in the current report “REGULATORY SYSTEM STRENGTHENING IN THE AMERICAS” (3) include:

- “The NRAs need to prioritize regulatory life cycle management of products, finding ways to better handling them and improving regulatory oversight using a holistic view of the entire life cycle of the authorization, improving the allocation of technical and human resources, and adopting electronic tools to improve efficiency.
- To implement procedures that enable the use of reliance.
- To improve publicly available regulatory information as part of good regulatory practices.
- To take advantage of available tools on GMP information. Make better use of public databases, such as EudraGMDP and WHO prequalification databases, to check the GMP status of individual manufacturing sites.
- Trading of integration mechanisms can facilitate regulatory strengthening.
- To develop legal and organizational frameworks.”

Agreements signed among NRAs of Latin American countries and NRAs should be considered as the main element of any legal frameworks.

### Recommendations

The common mission between the Industry and NRAs is to ensure that the available therapies should satisfy the patient's needs on time with optimal levels of quality, safety, and efficacy.

It is crucial to emphasize the importance of adjusting regulations to optimize the efficiency of the processes related to the PACs of drug (medicinal), biological, and biotechnological products by allowing the following:

1. the implementation of ICH Q9, Q10, Q12 principles based on QRM and effective Quality Management Systems, so that only the most significant PACs are submitted to regulators for prior approval,
2. the standardization of the reporting classifications of PACs with those of the Stringent Regulatory Authorities, so that the implementation of PACs is consistently and timeously carried out by manufacturers,
3. the setup of defined timelines for reviewing and approving major PACs, not exceeding 6 months, and
4. the development of reliance and Recognition processes as regulatory pathways.

There should also be built-in contingencies for the possibility of fast-tracking as required, not only to face emergencies like the COVID-19 pandemic but also to allow the continuous improvement required to guarantee product quality, safety, and efficacy.

There are some agreements between some National Regulatory Agencies in Latin America with SRA, like the one recently signed between EMA and ANVISA, the Confidential Agreement signed between the Americas'NRAr (27), and the agreements signed between México and EMA, FDA, Switzerland, Australia and Canada, which can be referred to while implementing this proposal and local regulations are updated.

NRAs of Latin America should implement and optimize their digital platforms if possible. Data collection and metrics related to PACs should be evaluated annually to determine the efficiency of the different measures, and to identify the changes and improvements that need to be implemented in processes and procedures.

Additionally, the Latin American agencies should also define a target timeline for PAC approval and performance indicators to improve efficiency.

This document provides the industry with views on the “Key Principles” documents and gives a holistic vision of what is needed to deliver timely and easily accessible medicinal products.

## Author Contributions

All authors designed the paper, analyzed the data, and wrote the manuscript.

## Author Disclaimer

The views expressed in this research paper are the independent views of the authors and should not be understood or quoted as being made on behalf of or reflecting the position of their respective companies or any other affiliation.

## Conflict of Interest

The authors declare that the research was conducted in the absence of any commercial or financial relationships that could be construed as a potential conflict of interest.

## Publisher's Note

All claims expressed in this article are solely those of the authors and do not necessarily represent those of their affiliated organizations, or those of the publisher, the editors and the reviewers. Any product that may be evaluated in this article, or claim that may be made by its manufacturer, is not guaranteed or endorsed by the publisher.

## Abbreviations

ANMAT, National Administration of Drugs, Food: and Medical Devices-Argentina
ANVISA: National Health Surveillance Agency-Brazil
COFEPRIS: Federal Commission for Protection against Health Risks-México
CMC, Chemistry, Manufacturing: and Controls
CPP: Critical Process Parameters
EMA: European Medicines Agency
FDA: United States Food and Drug Administration
GMP: Good Manufacturing Practices
ICH: International Council for Standardization of Technical Requirements for Pharmaceuticals for Human Use
INVIMA: National Food and Drug Surveillance Institute-Colombia
ISP: Public Health Institute-Chile
LATAM: Latin America
NRA: National Regulatory Agency
NRAr: National Regulatory Agency of Reference
PAC: Post-approval change
PAHO: Pan American Health Organization
PIC/S: Pharmaceutical Inspection Co-operation Scheme
QA: Quality Assurance
QC: Quality Control
QMS: Quality Management System
QRS: Quality Risk System
QTPP: Quality Target Product Profile
SRA: Stringent Regulatory Agency
WHO: World Health Organization
WLA: WHO Listed Authorities.
